# Morbidity Parameters Associated with Gastrointestinal Tract Nematodes in Sheep in Dabat District, Northwest Ethiopia

**DOI:** 10.1155/2018/9247439

**Published:** 2018-02-18

**Authors:** Zewdu Seyoum, Kalkidan Getnet, Mersha Chanie, Samuel Derso, Shumye Fentahun

**Affiliations:** ^1^College of Veterinary Medicine and Animal Sciences, University of Gondar, P.O. Box 196, Gondar, Ethiopia; ^2^Bureau of Dabat District Livestock and Fishery Development, Dabat, Ethiopia

## Abstract

Gastrointestinal nematode (GIN) infections of sheep and their interaction with selected morbidity parameters were studied in smallholder farms. 120 faecal samples were collected and examined using faecal flotation to determine nematode infection in sheep. Thus, the study demonstrated overall prevalence of 57.5% GIN infections, while the mean faecal egg count (FEC) was 517.5 EPG. The severity of GIN infection was determined based on EPG as a mild infection (EPG <500), 55.1%, moderate infection (EPG = 500–1500), 30.4%, and heavy infection (EPG >1500), 14.6%. Five genera of nematodes were identified using coproculture:* Haemonchus* (33.3%),* Trichostrongylus* (26.7%),* Bunostomum* (20%),* Oesophagostomum* (13.3%), and* Cooperia* (6.7%). A significant difference was observed in the mean FEC among the FC (*P* < 0.001), FAMACHA© score (*P* < 0.01), and the BCS of the animals (*P* < 0.001). FEC was positively correlated with the FAMACHA© score (FS), while FC and BCS were negatively correlated. Thus, FAMACHA© chart can suggest well the severity of nematode infections and can serve as a suitable on-farm tool to identify nematode-infected sheep and gives a guide to identify sheep that need to be treated with an anthelmintic.

## 1. Introduction

Ethiopia possesses one of the largest ruminant inventories, including more than 57 million heads of cattle and 58 million small ruminants [1, 2]. As compared to large ruminants, small ruminants have many advantages for smallholder farmers via fewer feed costs, quicker turnover, easy management, and appropriate size at slaughter [3, 4]. However, their productivity is still low compared to the population due to poor nutrition, diseases, and poor genetic makeup of the indigenous stock [1].

In small ruminants, gastrointestinal nematodes (GINs) infections represent important challenges in the tropical and subtropical regions [5, 6]. Infections with GIN affect the welfare of the animals and cause huge economic losses in livestock farming [7]. The most common GINs that affect small ruminants are* Haemonchus*,* Trichostrongylus*,* Ostertagia*,* Cooperia*,* Bunostomum*,* Oesophagostomum*,* Chabertia*, and* Nematodirus* [8]. There are potential threats associated with economic losses through lowered productivity, reduced animal performance and weight gain, retarded growth, a cost of treatment, and mortality [6, 9, 10].

GIN infections in ruminants (mainly in sheep) are of major importance in most African countries; however, their economic impact is greater in Sub-Saharan Africa, including Ethiopia, due to the availability of suitable agroclimatic factors for diversified vertebrate hosts and nematode species [11]. In Ethiopia, fragmented studies indicated the importance of nematodes as a cause of decreased production in domestic ruminants [5, 11, 12].

Small ruminants in North Gondar zone have a paramount importance to the livelihood of people and sheep are the dominant species in the highland areas. However, diseases often prevent them from attaining optimum productivity [13]. Thus, diseases and parasitism resistance should be considered as a criterion in a breeding selection, and the weak animals should be separated from the flock as far as possible [14]. Particular practical and economic applications, which could be used for the management and health of sheep flocks, should be developed. Some practical applications like body condition score (BCS) and FS could be used to develop more healthy flocks as well as to select animals in farms and in breeding programs. On-time proper diagnosis of diseases and malnutrition is a prerequisite to reduce losses in communal areas [14, 15].

Mucous membrane colour determination is one of the easiest ways to monitor the condition of the host and has proven effective as a diagnostic tool [16]. This can be done with an FAMACHA© chart that was developed as a diagnostic on-farm tool in South Africa. The chart provides farmers with a facility to identify animals that require anthelmintic treatment by comparing the ocular mucous membrane colour using a defined colour chart [14, 17]. The FAMACHA© chart categorises the anaemic status of small ruminants based on the conjunctiva mucosal colour on a scale from 1 (optimal eye colour, red) to 5 (pale eye colour, white) [17, 18].

Methods that can be employed in determining health and nutritional status of sheep include body weights and condition changes, worm burdens, and the FAMACHA© technique [19]. Body condition scoring (BCS), which is simple and is easily applied in clinical scoring by touching the tissue over the lumbar vertebrae, appears to be promising for this purpose [14]. The use of BCS and body weights in health and nutritional status management has been reported by researchers [14, 19]. However, in sheep, there is a high genetic correlation with FAMACHA© scores, haematocrit values, and faecal egg counts (FECs) [20]. This implies that other methods of health and productivity management in sheep should be developed to increase the efficiency of identification of sheep in poor health. One such option is the FAMACHA© system [21] which was developed in South Africa for classifying sheep into categories based on different levels of anaemia [22]. The practicability of this technique has been confirmed in on-farm application [21]. The effectiveness of the technique has been evaluated in the identification of parasite-resilient and/or resistant breeding rams and thus identification of stud rams with greater resilience/resistance to gastrointestinal parasites [23]. Moreover, the use of this system as part of an integrated control approach to nematodes in sheep kept by resource-poor farmers is recommended. Although the FAMACHA© system is useful in predicting the health status of sheep, it cannot be used in isolation, since it only detects the presence of* Haemonchus* in a sheep flock. It, therefore, implies that it should be coupled with other methods such as body condition score in predicting the condition of the sheep. But farmers have no knowledge and experiences about how to evaluate whether the sheep are infected with parasitic disease or not and no attempts have been made to identify morbidity parameters that would be valuable as a diagnostic tool for nematode infections in domestic animals. Therefore, the objectives of the current study were (1) to investigate the relationship between GIT nematode infections and associated parameters such as BCS and FS and (2) to evaluate the value of the FAMACHA© method for detecting anaemia in sheep reared in extensive farm conditions.

## 2. Materials and Methods

### 2.1. Study Area

This study was carried out from December 2015 to March 2016 in Dabat district, North Gondar Zone of Amhara Regional State, Northwest Ethiopia. The district is 814 km away from Addis Ababa. It is located at 12°59′03′′N latitude and 37°45′54′′E longitude at an altitude of 1500–3200 meters above sea level. The mean annual temperature in the district ranges between 18°C and 35°C. The mean annual rainfall of the district ranges from 800 to 1400 mm with a bimodal pattern. The long rainy season starts from the end of May and ends during September, and the short rainy season extends from March to April. The land is characterized by plain plateau and covered by various bushes and by some semihumid and humid highland vegetation. The farming system in the area is mixed livestock and crops, and sheep are the 2nd dominant animal species reared by farmers. The total sheep population comprises more than 80,000 heads of animals [24].

### 2.2. Study Animals

The study population consisted of sheep, which are kept under an extensive management system by smallholder farms within the highland agroclimatic zone. The study animals were female sheep that have an age above 6 months up to 2 years as inclusion criteria. Similarly, the animals did not receive anthelmintics before three months of sampling.

### 2.3. Study Design and Sampling Method

A field follow-up study was conducted on a total of 120 female sheep from three purposefully selected sites. A systematic random sampling method was employed to identify the study animals from each selected flock. Faecal samples from individual animals were collected per rectum using clean, labelled plastic bags. FAMACHA© chart score, faecal consistency, and body condition score were determined for each animal at the sampling time. Samples were obtained at intervals, which involve at least one visit per month for three months.

### 2.4. Parasitological Examinations

#### 2.4.1. Faecal Egg Counts

The faecal samples were collected from the rectum of selected animals and were brought to the Veterinary Parasitology Laboratory, Faculty of Veterinary Medicine, University of Gondar. Faecal egg count was determined by a modified McMaster technique using the saturated salt solution as a flotation fluid to quantify nematode ova [25]. The severity of GIN infection was assessed by description as a mild infection (EPG < 500), moderate infection (EPG = 500–1500), and heavy infection (EPG > 1500) [26]. In addition, faecal samples containing nematode eggs were pooled per each visit. The identification of the gastrointestinal nematode genera was made based on the morphology of 3rd stage larvae recovered from a coproculture of infected pooled faecal samples [27, 28].

### 2.5. Body Condition Scoring

Body condition scores (BCS) were determined by physically feeling the level of muscling and fat deposition over and around the vertebrae in the loin region. This was categorised on a scale of 1–5, according to Friedricks [29], where a score of 1 indicates thin and emaciated sheep, while a score of 5 indicates obese sheep.

### 2.6. FAMACHA© Scoring

The FS were determined by opening the lower eyelid of the sheep prior to and comparing the colour of the conjunctiva with five different scores on the chart, where 1 indicates nonanaemic sheep, while 5 indicates severely anaemic sheep, as described by Kaplan et al. [21].

### 2.7. Faecal Consistency

The FC was also scored as a pellet (1), medium/smooth (2), or soft/watery (3) and an average faecal consistency score was calculated based on Dorny et al. [19].

### 2.8. Data Management and Analysis

The collected raw data and laboratory results were coded and entered into MS excel spreadsheet. All statistical analysis was conducted using SPSS version 20. Parameters were analysed by one-way analysis of variance (ANOVA). *χ*^2^ test was also used to measure the association between nematode infection and faecal consistency, FAMACHA© score, and body condition score. Moreover, correlations between variables were determined by Pearson's correlation. In the analysis interpretation, the confidence level was held at 95% and the result was considered to be significant when* P* < 0.05.

## 3. Results

### 3.1. Association between Body Condition Scores and Nematode Infection

**Figure 1:**
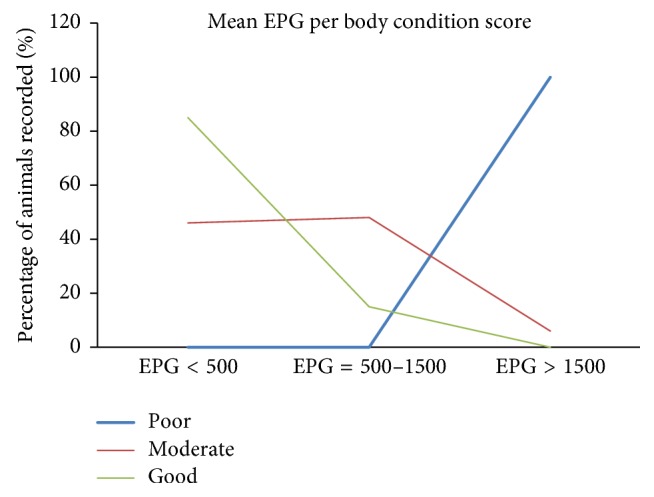
Body condition score with mean EPG.

Gastrointestinal nematode infection in examined sheep showed significant association with a body condition score (*P *< 0.05). Poor body condition scored animals had a higher proportion of nematode infection than moderate and good body condition scored animals. However, faecal egg count showed a strong negative correlation with the BCS (*r* = −0.72,* P* < 0.001). Sheep that had the lowest mean FEC value of 127.98 ± 232.06 had good BCS, while sheep that had the highest mean FEC value of 2373.33 ± 461.83 had poor BCS (Table 2 and Figure 1). Moreover, the mean FEC values of the different BCS were significantly different from each other (*P* < 0.05).

### 3.2. Association of Faecal Consistency (FC) and Nematode Infection

**Figure 2:**
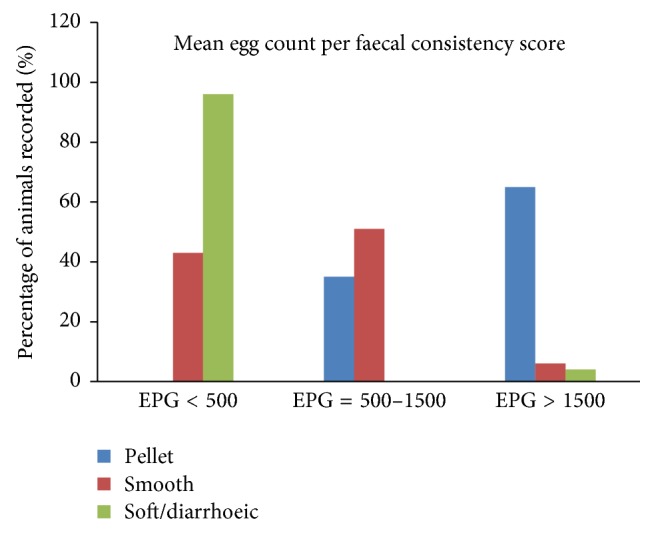
Faecal consistency score with mean EPG.

The overall prevalence of GIN infection was significantly different (*P* < 0.05) among faecal consistency. Higher prevalence was recorded in animals that defecated pellet and smooth faecal matter than in animals with the soft/diarrhoeic faecal matter. A significant association was also noted in the mean egg count among the faecal consistency (*F* = 90.279,* P* < 0.001). However, there was a negative correlation between FC and FEC (*r* = −0.72,* P* < 0.001). Sheep that had higher mean FEC value of 1906.25 ± 712.560 had pellet FC, while sheep with lower mean FEC value of 105.66 ± 289.62 had soft/diarrhoeic FC (Table 2 and Figure 2).

### 3.3. Association between FAMACHA© Scores and Nematode Infection

**Figure 3:**
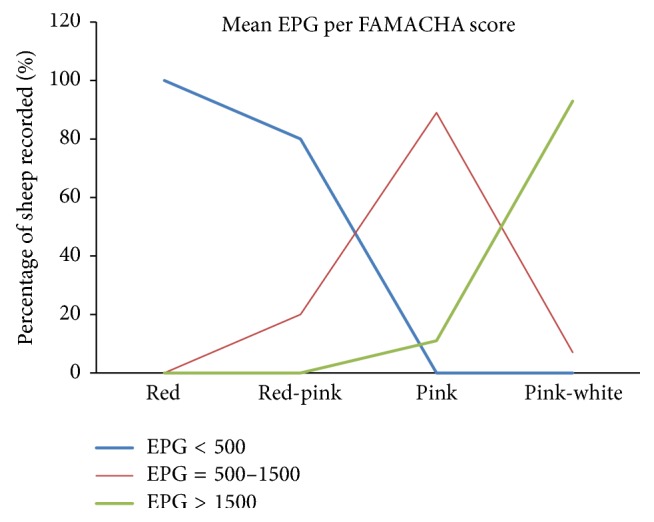
FAMACHA© score with mean EPG.

A strong positive correlation (*r* = 0.85,* P* < 0.001) was noted between the FAMACHA© score and the faecal egg count. The proportion of sheep with 2, 3, and 4 FAMACHA© scores was significantly (*P* < 0.05) increased as the faecal egg count increased (Table 1). Similarly, animals with pink and pink-white ocular conjunctiva had a higher mean egg count than animals with red and red-pink ocular conjunctiva. Moreover, animals with pink and pink-white conjunctiva colour had a higher GIN infection rate than animals with red and red-pink conjunctiva colour (Table 1 and Figure 3).

### 3.4. Faecal Culture

The results of coproculture revealed that* Haemonchus* was the dominant nematode, representing 33.3% of the total larval recovery from cultures.* Trichostrongylus* species were the next most prevalent nematode, representing 26.7% of the total infective larvae harvested. Others, including* Bunostomum*,* Oesophagostomum*, and* Cooperia* species (in order of dominance), were found in varying percentages, representing 6–20% of the larval cultures.

## 4. Discussion

Gastrointestinal nematodes are responsible for substantial losses of productivity in the livestock industry. In small ruminants, GINs can result in anaemia due to the haematophagous activities of nematodes, diarrhoea because of gastroenteritis or digestion/absorption disruption effects, and chronic weight loss and weakness due to the depression of appetite and reduction of feed digestibility. All these effects result in serious economic losses to the producer and the nation in general. The prevalence of GINs seen in the current study was relatively high compared to a previous report on the extensively managed system [30, 31]. This could be due to the availability of suitable climatic conditions that support the prolonged survival and development of an infective larval stage of most nematodes [32]. Similarly, this might be due to a communal pasture system that allows other sheep to graze on the same pasture. Similar circumstances might be possible for the high prevalence.

In the present study, animals with poor body condition score showed significantly higher mean parasite egg count. This agrees with the findings of Sani and Gray [33], Nigatu [34], and Kanyari et al. [35]. Moreover, the present report agrees with the finding of Sissay et al. [5] who described the effect of nematode infection on the mean body weight gain of the studied sheep. This poor body condition might be due to malnutrition or other concurrent bacterial and parasitic infections, which lead to a poor immunological response to infective stage of the parasites. This results in higher susceptibility to infection than other groups. The production of eggs by the nematodes is also dependent on the immune status of the host as the innate immunity can also suppress egg production if the animal is in good health status; thus nutrition and the level of protein feeding play a major role in nematode managing.

According to the present study, the FAMACHA© chart is an effective method and can be used by smallholders as a diagnostic tool to identify animals exposed to nematodiasis. The chart result was correlated with the faecal egg count. Sheep with higher EPG also had high FS, which strongly suggests that worm burdens were the main contributory factor to anaemia and the FAMACHA© score had a strong correlation (*r* = 0.85) with EPG; that is, the proportion of sheep with 2, 3, and 4 FAMACHA© scores increased as the EPG increased. This is in agreement with the report of Sissay et al. [5] who confirmed that the FAMACHA© score had a strong correlation (*r* = 0.8,* P* < 0.001) with EPG. These findings may be explained by the presence of higher number of blood-sucking nematodes (like* Haemonchus* species). Blood-sucking nematode infection can cause anaemia, diarrhoea, and loss of weight and appetite as the parasites suck blood from the mucosal membrane of the intestine, resulting in irritation and swelling of the intestinal lining. The parasite may also cause loss of nutrients when competing for nutrients with the host. Therefore, damaging the mucosal membrane of the intestine could result in malabsorption, impaired digestion, and protein loss [36]. In addition, blood loss and completion of nutrients result in an electrolyte and red blood loss. All such events result in an anaemic condition of the infected animals.

The present finding is also in agreement with earlier results obtained for Barbari and Jamunapari kids in Pakistan by Coop and Kyriazakis [37] and Chauhan et al. [38]. Although sheep are usually coinfected by several species of GI nematodes,* Haemonchus* species were the most prevalent small ruminant trichostrongylid. Daily blood loss caused by single adult* Haemonchus* has been estimated to be 0.05 ml [39, 40]. Also, female* Haemonchus* species have been reported to be highly fecund, with a single female releasing between 5,000 and 10,000 eggs in the faeces daily [41]. These factors explain the high positive correlation between FS and FEC observed in this study.

44% of faecal samples had gotten soft/diarrheic faecal consistency in this study. Interestingly, there was an inverse relationship between gastrointestinal nematode FECs and faecal consistency. Similarly, Ganaba et al. [42] reported the absence of an association between diarrhoea and FEC in a study on calf morbidity and parasite prevalence in Burkina Faso. This could be due to the fact that the consistency of the faeces may affect the number of eggs/gram of faeces markedly as the more watery the faeces are, the more diluted the eggs are.

Availability of 3rd larvae of GINs showed similar patterns to the nematode faecal egg counts. The observed result estimated the higher proportion of* Haemonchus* species L3 in faecal cultures, similar to study in other reports [6, 43]. This can be attributed to the variation in the biotic potential of nematodes and hence* Haemonchus* species rapidly take up dominance at times when environmental conditions on pasture are suitable for larvae development and survival in the external environment. In addition, overstocking, which is a major problem in many African communal grazing areas, may contribute to the availability of a large number of infective larvae of GI nematodes.

In conclusion, gastrointestinal nematodes are responsible for substantial losses of productivity in the livestock industry. Their harmful effects on small ruminants range from anaemia owing to the haematophagous activities of nematodes to diarrhoea because of gastroenteritis or digestion/absorption disruption effects and chronic weight loss/emaciation and weakness due to the depression of appetite and reduction of feed digestibility. All these clinical effects result in serious economic losses to the producer and the nation in general. In the present study, important helminth genera of sheep including* Haemonchus*,* Trichostrongylus*,* Bunostomum*,* Oesophagostomum*, and* Cooperia* were identified.* Haemonchus* was the most prevalent nematode, representing 33.3% of the recorded nematodes. This study also showed inevitably the use of morbidity parameters in examining nematode infections as seen by the high egg counts and FAMACHA© score (score of 3-4), body condition score (score of 1), and faecal consistency (score of 1). Overall, the animals appeared normal and healthy and this is the fact that farmers need to realise in their efforts to control nematodiasis that animals may buckle under very quickly if allowed to continue to have worm infections. Therefore, it is hoped that this report can be used to encourage farmers to use the FAMACHA© chart, body condition score, and the faecal consistency score, which can bring awareness to the importance of nematode infections in apparently normal animals. If the combination of morbidity markers is used correctly and consistently in a flock, such a highly susceptible animal could be easily identified and treated to reduce the contamination of pasture for the rest of the flock. Hence, community training on various methods to improve animal health management systems is essential. Provision of animal health extension services includes periodic surveillance of nematode infection, determination of anaemia using the FAMACHA© chart, and treatment of animals based on the outcome of these analyses.

## Figures and Tables

**Table 1:** Association between nematode infection and risk factors in sheep of Dabat district.

Variables	Number of animals	Number of infected animals (%)	*χ* ^*2*^	*P* value
Body condition				
Poor	10	10 (100)	17.677	0.001
Moderate	54	37 (68.5)		
Good	56	22 (39.3)		
Faecal consistency				
Pellet	16	16 (100)	26.426	0.001
Smooth/medium	51	35 (68.6)		
Soft/watery	53	18 (34)		
FAMACHA© score				
Red	20	7 (35)	30.296	0.001
Red-pink	70	32 (45.7)		
Pink	18	18 (100)		
Pink-white	12	12 (100)		

**Table 2:** One-way ANOVA show difference between mean faecal egg count and risk factors.

Parameters	No. animals	Mean ± SD	*F*	*P-*value
Body condition				
Poor	10	2373.33 ± 461.83^a^	111.955	0.001
Moderate	54	577.78 ± 577.21^b^		
Good	56	127.98 ± 232.06^c^		
Faecal consistency				
Pellet	16	1906.25 ± 712.56^a^	90.279	0.001
Smooth/medium	51	509.80 ± 526.21^b^		
Soft/ diarrhoeic	53	105.66 ± 289.62^c^		
FAMACHA^©^ score				
Red	20	51.67 ± 91.43^a^	202.015	0.001
Red-pink	70	188.10 ± 263.08^ab^		
Pink	18	352.48 ± 83.08^c^		
Pink-white	12	564.40 ± 162.93^d^		

*Note.* Different letters as suffix indicates the significant variation along the column in each considered factor.
